# Old Age Exacerbates White Matter Neuroinflammation and Cognitive Deficits Following Closed-Head Injury, Particularly in Female Mice

**DOI:** 10.1089/neur.2024.0074

**Published:** 2024-08-22

**Authors:** Teresa Macheda, Margaret R. Andres, Lydia Sanders, Kelly N. Roberts, Ryan K. Shahidehpour, Josh M. Morganti, Adam D. Bachstetter

**Affiliations:** ^1^ Department of Neuroscience, University of Kentucky, Lexington, Kentucky, USA.; ^2^ Spinal Cord and Brain Injury Research Center, University of Kentucky, Lexington, Kentucky, USA.; ^3^ Sanders-Brown Center on Aging, University of Kentucky, Lexington, Kentucky, USA.

**Keywords:** aging, astrocytes, brain injury, microglia, neuroinflammation, sex differences

## Abstract

The increasing incidence of traumatic brain injury (TBI) among older adults, particularly mild injuries from falls, underscores the need to investigate age-related outcomes and potential sex differences in response to TBI. Although previous research has defined an aging-TBI signature (heightened glial responses and cognitive impairment) in open-skull moderate-to-severe TBI models, it is unknown whether this signature is also present in mild closed-head injuries (CHIs). This study explores the influences of age and sex on recovery in a mouse CHI model induced by an electromagnetic impactor device in 4-month-old and 18-month-old C57BL/6 mice. We assessed the righting reflex, body weight, behavior (radial arm water maze and active avoidance), and inflammation (GFAP, IBA1, CD45) in the neocortex, corpus callosum, and hippocampus. We observed that aged female mice exhibited more severe TBI-induced cognitive deficits. In addition, a more pronounced reactive neuroinflammatory response with age was noted within white matter regions. Conversely, gray matter regions in aged animals either showed no enhanced pathological changes in response to injury or the aged mice displayed hyporesponsive glia and signs of dystrophic glial degeneration that were not evident in their younger counterparts following CHI. These findings suggest that aging influences CHI outcomes, partially reflecting the aging-TBI signature seen in more severe injuries in white matter, while a distinct aging and mild-TBI signature was identified in gray matter. The heightened vulnerability of females to the combined effects of age and mild CHI establishes a foundation for further investigation into the mechanisms underlying the sexually dimorphic response in aging females.

## Introduction

As life expectancy increases and the global population ages, the incidence of traumatic brain injuries (TBIs) in older adults is rising—a trend that cannot be ignored. TBIs, predominantly mild in severity due to falls, constitute nearly 80% of all TBIs in individuals older than 50. In this demographic, the term “mild” belies the true impact of the injury: up to 55% of these older adults suffer unfavorable outcomes—a significant increase from the 15% reported in younger populations.^1–6^ These injuries are more frequent and more severe in older adults, leading to extended hospitalizations, an increased likelihood (15–67%) of a transition to nursing homes, and elevated mortality rates.^2^ The long-term consequences are profound, with a heightened risk of post-traumatic epilepsy, delayed seizures, and a predisposition to neurodegenerative disorders such as dementia and Parkinson’s disease, with respective increased risks of 26% and 44%.^5^ Managing TBIs in this age group is further complicated by preexisting comorbidities, polypharmacy, and an increased potential for drug interactions.^1–6^

Women, who are particularly underrepresented in pre-clinical animal model studies despite accounting for 62% of injuries in those older than 85, face unique challenges.^3^ Previous research on TBIs in aged animals has predominantly featured male subjects or has not specified the sex of the animals, nor has it assessed the effects of sex on outcomes—a significant oversight given the potential for sexually dimorphic mechanisms to influence recovery trajectories and outcomes.^7–23^ Furthermore, the focus has been on moderate-to-severe TBI, which leaves a critical knowledge gap regarding the aged brain’s response to mild closed-skull injuries.

Our study addresses these gaps and advances the field by exploring whether the aging-TBI signature—marked by exacerbated glial changes and cognitive impairment observed in severe TBI models—is also present in mild TBI (mTBI) induced by closed-head injury (CHI) in male and female mice. We tested 18-month-old mice, equivalent to 60–70-year-old humans, and 4-month-old mice, analogous to 20–30-year-old humans.^24^ Our findings reveal that aged female mice suffer more pronounced TBI-induced deficits than their male counterparts and that white matter is particularly susceptible in older mice, with hyporesponsive and potentially dystrophic glia observed exclusively in the aged cohort postinjury. This study not only sheds light on the aging-TBI signature in a mild injury context but also underscores the urgent need for sex-specific research in the development of targeted therapeutic interventions.

## Materials and Methods

### Animals

The Institutional Animal Care and Use Committee of the University of Kentucky approved all experiments, which were carried out as per the Guide for the Care and Use of Laboratory Animals and ARRIVE guidelines. C57BL/6 adult mice (4–5 months old, 46♂/44♀, young group) and aged (18–20 months old, 45♂/42♀, aged group) mice were acclimated in our facility for at least a week before the experiment commenced and were then randomly assigned to groups before any manipulations or tests. Animals were housed in a controlled humidity and temperature environment with free access to food and water and kept on a 12/12-h (7 am–7 pm) light/dark cycle.

### Experimental design

**FIG. 1.**
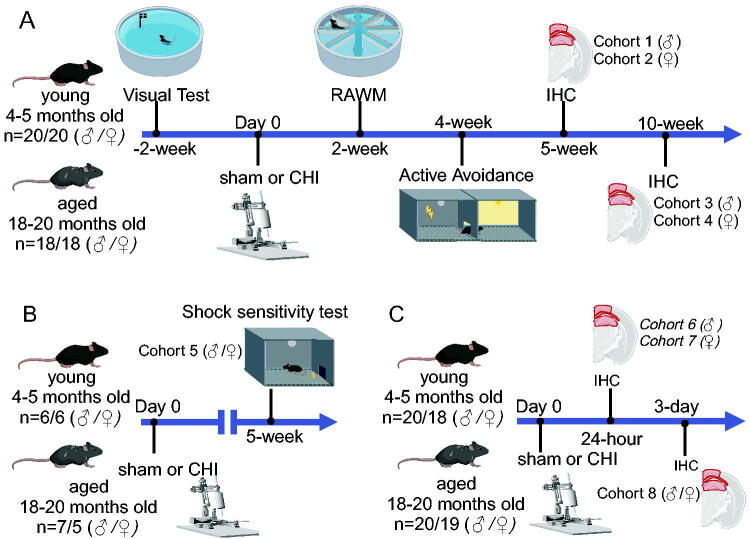
Experimental design. Eight cohorts of mice were studied. **(A)** In cohorts 1–4, male (cohorts 1,3) and female (cohorts 2,4), young and old mice underwent a behavioral battery. This included visual acuity screening in an open pool (2 weeks before surgery), using an open pool without extra-maze cues. Cognitive testing involved an 8-arm RAWM at 2 weeks postinjury and active avoidance testing at 4 weeks postinjury. Mice were then euthanized at either 5 or 10 weeks postinjury for brain immunohistochemistry (IHC) analysis. **(B)** Cohort 5 mice were evaluated for shock sensitivity 5 weeks postinjury. **(C)** Mice in cohorts 6–7 were sacrificed 24 h postsurgery and cohort 8 at 72 h, with brains collected for IHC.

A block design was used, ensuring equal representation of both age groups (young and aged) and injury types (sham and CHI) within all cohorts, with the order of the mice randomized within the 8 total cohorts. For behavioral assessment, the mice were stratified by sex, with male and female subjects being tested in separate cohorts. Cohorts 1–4 were behaviorally tested. First, the mice were screened for visual and motor impairment; no mice failed these tests. Following sham or CHI surgery, the mice were tested in the radial arm water maze (RAWM) at 2 weeks postinjury, with active avoidance behavior at 4 weeks postinjury, and euthanized at 5 weeks (cohorts 1–2), or 10 weeks (cohorts 3–4) postinjury for immunohistochemistry (IHC) (Fig. 1A). An independent cohort of mice (cohort 5) was used in the shock sensitivity test (Fig. 1B). The active avoidance assay applied this specific shock intensity (Fig. 1B). Cohorts 6–7 and 8 were used for IHC analysis at 24 and 72 h postinjury, respectively (Fig. 1C).

### CHI procedure

The mice received the CHI procedure following the protocol described in Macheda et al.,^25^ using a digital stereotactically guided electromagnetic impactor device (Leica Biosystems). For surgery, mice were anesthetized with 4-3.5% isoflurane in 100% O_2_ and maintained under anesthesia with continuous inhalation of isoflurane (2.5-3.5%, 1L/min) in 100% O_2_ through a nose cone during surgery. While in the stereotaxic frame, a midline sagittal scalp incision was made to expose the coronal and sagittal sutures of the skull. Then, a 1 mL latex pipette bulb filled with water was placed under the mouse head. Then, a single controlled midline impact was administered at a controlled velocity of 5.0 ± 0.2 m/s with a dwell time of 100 ms, using a 5.0 mm flat steel tip, at the following coordinates: mediolateral, 0.0 mm; anteroposterior, 1.5 mm; and 1.0 mm deep. Sham-operated mice went through the same procedure as CHI mice, but no impact was delivered. Among the animals that underwent surgery, two mice died or were excluded because of surgical complications, one from the aged+CHI and one from the young+CHI groups.

### Behavioral assays

All behaviorally tested mice were handled 3 days before the start of the experiment and moved to the behavioral testing room at least 20 min before the test. Behavioral testing was conducted between 8 am and 4 pm, and the testing personnel were blinded to the experimental groups.

#### Visual acuity test

The visual acuity test assessed visual discrimination and acuity in mice before surgery (cohorts 1–4). Mice were tested in a water pool with a diameter of 121 cm that was isolated from the rest of the room by double black curtains. A circular platform (diameter = 8 cm) was submerged 1 cm below the water level and made visible by a flag. The water was made opaque by adding nontoxic paint (Sax 2684 Versatemp Non-Toxic Heavy Body Tempera Paint), and the water temperature was held constant at 22 ± 1°C. The light intensity in the pool area was 4–6 lux, and no extra-maze cues were used during this test. The pool was divided into four quadrants (South -S-, West -W-, East -E- and North -N-), with the platform in the SW quadrant. The test comprised four trials, and the mice had up to 60 sec to locate the platform. The drop location (E, W, S, or N) varied between trials in a semirandom manner. The mice were allowed to rest and recover between trials in a holding cage placed half on a heating pad. EthoVision XT 11.0 (Noldus Information Technology) was used to record and score the mice behavior (latency to reach the platform, total distance, and speed). All mice met the vision criteria of finding the platform within 60 sec across the four trials.

#### Radial arm water maze

The mice were tested in the 8-arm RAWM at 2 weeks post-CHI or sham surgery following the protocol in Macheda et al.^26^ Briefly, mice were tested in a total of 28 trials over the 4-day test, with days 1 and 2 designated as training days because the escape platform was made visible by a flag on trials 1 and 3, days 3 and 4 as testing days, and the platform was hidden during all the trials. To prevent learning limitations and fatigue, the 7 trials were split into two blocks, with 9–10 mice completing a block before the next group undertook the next block in a staggered design. Two aged+CHI mice (1 male, 1 female) were excluded from the assay due to impaired swimming ability. The behavior was recorded using EthoVision XT 11.0 (Noldus Information Technology) to score the total number of errors, latency to escape, distance traveled, and velocity.

#### Shock sensitivity test

The mouse pain threshold to shock was tested in a two-way shuttle box (Gemini, San Diego instruments). This test was performed 5 weeks postsurgery following a detailed protocol outlined in Macheda et al.^27^ The mouse was placed in one of the two compartments of the shuttle box and exposed to a series of 2-sec foot shocks with gradually increasing amperage at 30-sec intershock intervals after a 120-sec acclimation period. The range of shock intensity used varied from 0.01 to 0.30 mA. Response to the foot shock was evaluated using a scale that ranged from 0 (lowest score) to 4 (highest score).

#### Active avoidance rest

At 4 weeks postinjury, the active avoidance test was conducted using the two-way shuttle box (Gemini, San Diego instruments) following the protocol described in Macheda et al.^27^ In brief, mice were trained over 5 days to avoid a mild foot shock (unconditioned stimulus; 0.2 mA) by associating it with a conditioned stimulus (light) in 50 trials per day. The latency and number of successfully avoided trials were recorded and analyzed. In the active avoidance paradigm, mice meeting the exclusion criteria consistently evaded the unconditioned stimulus on the first training day and did not experience a foot shock. In our study, both aged and young mice and sham and CHI groups demonstrated proficient performance in the assay.

### Immunohistochemistry analysis

Mice were deeply anesthetized with 5% isoflurane in 100% O_2_ and then transcranially perfused with ice-cold 1× phosphate-buffered saline for 5 min. The brains were rapidly removed. Left hemibrain was fixed in 4% Paraformaldehyde for 24 h, and then transferred in 30% sucrose. The left hemibrain was coronally sectioned in 30-µm-thickness sections using a microtome. Sections were stored at −20°C in cryoprotectant until used for IHC analysis. Mice were sacrificed at 24 and 72 h, at 5 and 10 weeks postsurgery.

As previously described, IHC staining was completed on free-floating sections following established methods.^28,29^ Every 10th section between 1.3 mm and 2.5 mm was included in the IHC analysis. The primary antibodies used for the staining were as follows: (1) rabbit anti-glial fibrillary acidic protein (GFAP) (1:10,000 Dako, catalog no. Z0334 RRID: AB_10013382) for astrocytes, (2) rabbit anti-ionized calcium binding adapter molecule 1 (IBA1) (1:10,000, Wako, catalog no. 019–19741) for microglia, (3) rat anti-dectin1 (InvivoGen, catalog no. mabg-mdect, RRID: AB_2753143) for disease-associated microglia, and (4) rat anti-CD45 (1:1,000, BioLegend, catalog no. 103102, RRID: AB_312967) for microglia/macrophages and lymphocytes. The entire slide was scanned using the Zeiss AxioScan Z.1, and HALO software (Indica Labs Inc., Albuquerque, NM) was used to quantify the specific staining volume after the region of interest (neocortex, corpus callosum, and hippocampus) was outlined. For GFAP, IBA1, and CD45, the HALO area fraction algorithm quantified specific staining in the region by normalizing the number of positive pixels to the outlined area, accounting for variations in regional size. All slides in the batch were analyzed using consistent parameters, and color markup analysis was confirmed for each slide. An observer blind to treatment groups conducted all quantifications. Dectin-1 staining was sparse or absent on most slides. Sparse staining in the corpus callosum was initially observed following the CHI. In some animals, staining appeared in both the cortex and corpus callosum. Staining was never found in the cortex without also being present in the corpus callosum. Therefore, staining assessments were conducted by an observer blind to the experimental conditions, using a 5-point scale to evaluate the severity of changes in Dectin-1 staining. The scale is defined as follows: 1 = none, 2 = sparse in corpus callosum, 3 = dense in corpus callosum, 4 = sparse in both cortex and corpus callosum, and 5 = dense in both cortex and corpus callosum.

### Statistical analysis

Statistical analysis was performed using JMP Pro version 17.0 (SAS Institute, Cary, NC). Data visualization was done using GraphPad Prism version 10.0.2 or R version 4.3.0. Data are presented as mean ± standard error of the mean (SEM). Histological data were normalized for batch effects and plotted as fold change from the young sham area, as specified in the figure legend. Individual mice are represented in scatter plots, with the total number of mice used in the experiment indicated in Figure 1A and specified in the figure or figure legend. All data were stratified by sex before analysis. Statistical significance was determined at a *p* value < 0.05. The righting reflex was analyzed using an unpaired *t-test*. Body weight changes and behavioral assays were analyzed using a mixed-model approach with repeated-measures analysis of variance (ANOVA) and the standard least-squares method. For the shock sensitivity test, we used a chi-square at each level of shock intensity, as we wanted to know if there were any possible differences in shock sensitivity and at what level of shock intensity, and not main effects for age or injury. GFAP, IBA1, and CD45 histological data were analyzed using a two-way ANOVA after stratifying the data by postinjury time point. The area of CD45 staining was transformed to a Log2 scale before the two-way ANOVA to adjust for normality. Group differences were assessed using a Tukey *post hoc* test. Dectin-1 staining was analyzed using a two-way logistic regression, followed by a chi-square test for *post hoc* analysis.

## Results

### Age influences CHI-induced body weight changes, with sex-specific differences in severity and recovery patterns. The righting reflex is independent of age

**FIG. 2.**
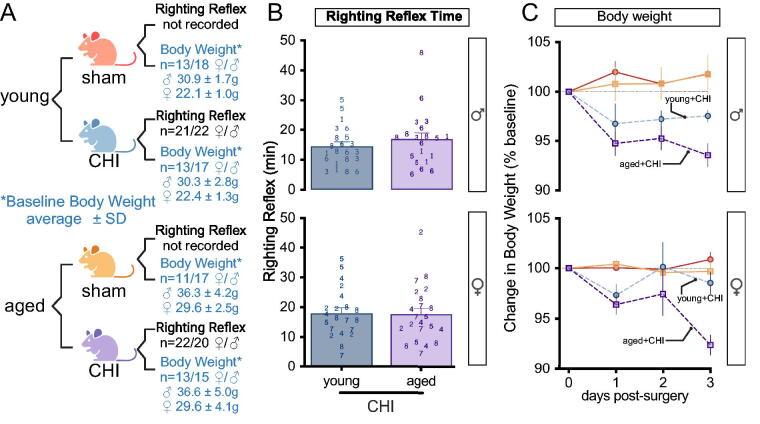
Righting reflex and closed-head injuries (CHI)-induced weight changes**. (A)** No significant differences in baseline body weight were found between CHI and sham groups. Expected age and sex differences were observed (grams, mean ± SD). **(B)** Righting reflex recovery time post-CHI was similar across all groups and cohorts (cohort number indicated). Sample size in (A). **(C)** CHI induced significant weight loss (*p*_(injury)_ < 0.01; repeated-measures ANOVA). This loss was observed in young and aged male mice, as well as aged females. Cohorts 6–7 were excluded due to the 24-h sacrifice. Data presented as mean ± SEM. Numbers in (B) indicate the cohort number.

Age and sex did not affect the righting reflex duration in mice post-CHI (Fig. 2A–B). Aged mice initially weighed more than young mice (*p*_(age)_ < 0.0001; two-way ANOVA) (Fig. 2A), but there was no difference by injury or injury-by-age interaction, supporting that the groups were balanced. In females, CHI-induced weight loss was observed over the first 3 days postinjury (*p*_(injury)_ = 0.002; repeated-measures ANOVA), with the most pronounced weight loss in aged+CHI mice compared with sham mice (*p* = 0.0027; Tukey test). Young+CHI mice did not differ significantly from their sham counterparts (Fig. 2C). Male mice also exhibited CHI-induced weight loss over the first 3 days (*p*_(injury)_ < 0.001; repeated-measures ANOVA). Unlike females, both young+CHI (*p* = 0.0089; Tukey test) and aged+CHI (*p* = 0.0085; Tukey test) male mice lost more weight than their respective shams.

By 5 weeks postinjury, female mice showed no effect of CHI on body weight. However, male mice still exhibited injury-induced body weight changes (*p*_(injury)_ = 0.006), with aged+CHI mice weighing less than aged+sham mice (*p* = 0.044; Tukey test) (young+sham (female: 102.3 ± 2.0, male: 102.6 ± 1.4); young+CHI (female: 104.9 ± 2.0, male: 99.1 ± 1.6); aged+sham (female: 96.6 ± 2.3, m: 95.4 ± 1.4); aged+CHI (female: 94.9 ± 2.3, male: 89.6 ± 1.4); mean percent of baseline ± SEM. By 10 weeks postinjury, no injury or age-by-injury interaction was observed for female or male mice.

### CHI-induced cognitive impairments in the RAWM at 2 weeks postinjury intensify with aging, especially in female mice

**FIG. 3.**
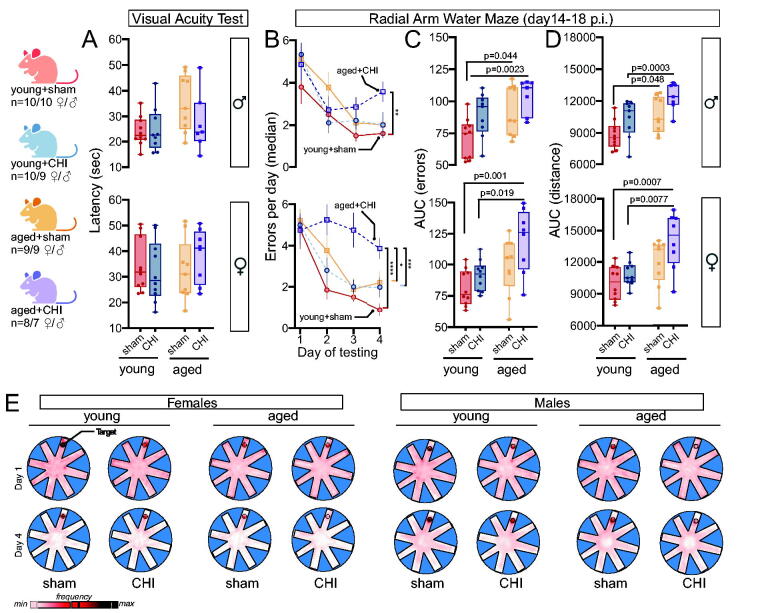
Closed-head injury (CHI) worsens age-related decline in spatial memory at 2 weeks postinjury in the radial arm water maze, particularly in female mice. **(A)** Pre-CHI visual acuity tests confirmed comparable task completion times across all experimental groups. **(B)** The radial arm water maze task revealed age-related performance deficits (*p*_(age)_; female = 0.0006, male = 0.019; repeated-measures ANOVA). In females, CHI also had a significant main effect on errors (*p*_(injury)_ = 0.007), while this effect was not statistically significant in males (*p*_(injury)_ =0.227; repeated-measures ANOVA). Notably, aged+CHI females made the most errors across the four days of testing, exceeding other female groups in both median errors and area under the curve (AUC) **(C)** and distance traveled **(D)** (Tukey test). Male mice exhibited a similar pattern, although the impact of age and injury was less severe. **(E)** Heatmaps illustrate mean group-specific visitation frequencies (pink=lower, black=higher). Data from Days 1 and 4 demonstrate a common learning trajectory; initially, mice displayed higher exploratory behavior, which decreased over time as they successfully located the target (indicated by a darkening of the target area). Young sham mice showed the most pronounced learning curve by Day 4. Data are presented as average ± SEM, with each point representing an individual animal.

Existing research shows that aged mice with moderate-to-severe TBIs have greater cognitive impairments than younger mice.^11,13,18,19,22,23^ We investigated whether this trend applies to recovery from mild CHIs in aged mice. Initially, we tested visual acuity two weeks before the injury to ensure that mice with visual impairments or swimming difficulties were reassigned to a nonbehavioral testing cohort. Before the injury, all mice were able to complete the task. Furthermore, randomly allocating mice to the sham or CHI groups did not cause imbalances in task performance abilities (Fig. 3A). As anticipated, aged mice swam slower than younger mice. Therefore, the RAWM task scores were based on incorrect arm entries (errors) and distance, which are less influenced by age.

Starting two weeks postinjury, mice underwent four days of RAWM testing. In females, both CHI and older age were associated with increased task errors (*p*_(injury)_ = 0.007; *p*_(age)_ = 0.0006; repeated-measures ANOVA). For males, only age was associated with more errors (*p*_(age)_ = 0.019; repeated-measures ANOVA) (Fig. 3B). Both female and, to a lesser extent, male mice demonstrated impaired task learning, showing similar error rates on the final day as on the first. Aged+CHI mice made the most errors; specifically, female aged+CHI mice made more errors than young+CHI (*p* = 0.011; Tukey test) or young+sham mice (*p* = 0.0003; Tukey test).

Analysis of area under the curve (AUC) for errors (Fig. 3C) and distance traveled (Fig. 3D) further confirmed worse performance with age and injury, as indicated by a larger AUC in both sexes (Fig. 3C: *p*_(age)_ male = 0.004, female = 0.0009; *p*_(injury)_ male = 0.015, female =0.021; Fig. 3D: *p*_(age)_ male = 0.0008, female = 0.0003; *p*_(injury)_ male = 0.004, female = 0.04; two-way ANOVA). Heatmaps depicting average group paths on days 1 and 4 highlight this distinction. Young mice focused their searches around the escape platform, whereas older mice, particularly those aged+CHI, exhibited a broader, more scattered search pattern (Fig. 3E). Overall, aged mice performed worse than younger mice, with injury worsening this effect, particularly in females. We did not find an age-by-injury interaction for RAWM deficits.

The significant weight loss observed in old mice post-CHI may weaken the animals and potentially affect their motor performance in behavioral tests such as the RAWM. Our data show that at 5 weeks postinjury, male aged+CHI mice, but not female aged+CHI mice, still weighed less than their aged+sham counterparts. While we do not have body weight measurements at the 14–18-day time point when the RAWM was completed, it is possible that the overall health of the aged mice could contribute to observed deficits. This highlights the need for future studies to monitor and statistically evaluate changes in body weight from baseline as a potential mediating factor in behavioral results. The RAWM test we used minimizes the effects of motor impairments on performance assessment by focusing on errors rather than swimming speed or latency to find the platform. However, two aged+CHI mice were excluded due to impaired swimming ability, possibly influencing our results and underestimating the degree of impairment seen in aged mice following CHI.

### Cognitive impairments in active avoidance, induced by CHI, become more pronounced with aging, particularly in female mice

**FIG. 4.**
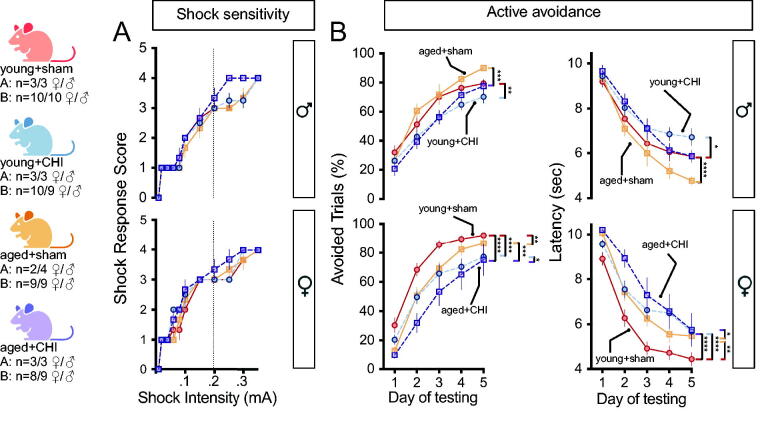
Impact of injury on associative learning in the active avoidance task. **(A)** Mice showed similar sensitivity to a 0.2 mA foot shock (the intensity used in the active avoidance task) regardless of age or injury. However, at 0.25 mA, aged+CHI mice of both sexes exhibited heightened sensitivity (hyperalgesia) compared with other groups (male *p*_(age)_ < 0.006; female *p*_(injury)_ < 0.038; ChiSq). **(B)** CHI significantly impaired active avoidance task performance at 4 weeks postinjury in both sexes, as seen in the lower percentage of shocks avoided and increased latency to respond (*p*_(injury)_; avoided < 0.01, latency < 0.01; repeated-measures ANOVA). In females, age was also associated with worse performance (*p*_(age)_; avoidance = 0.017, latency = 0.014; repeated-measures ANOVA). No age-by-injury interaction was observed for either sex. Data are presented as average ± SEM. CHI, closed-head injury.

The mice were then tested at 4 weeks postinjury in active avoidance behavior, which relies more on associative memory for task completion.^27^ The initial step involved determining whether aging or injury altered the animal’s sensitivity to the electric shock stimulus. At the standard shock intensity of 0.2 mA used in the avoidance behavior, no difference in shock response was observed between the groups, while at a shock intensity of 0.25 mA, female and male aged+CHI mice demonstrated higher shock response scores than aged+sham mice (female: *p* = 0.088; male: *p* = 0.025; ChiSq) (Fig. 4A). This suggests that brain injury, especially in older animals, might induce hyperalgesia, as observed in other TBI models,^30^ although not previously reported in the CHI model.^27^

After a CHI, male mice of all ages showed poorer performance in the active avoidance task than their same-age sham counterparts (Fig. 4B) in avoided trials and the latency to avoid/escape the shock (*p*_(injury)_: avoided = 0.0025, latency = 0.007; repeated-measures ANOVA). The young+CHI males and not the aged+CHI males were the worst performing group, yet the difference was not statistically significant.

In females, performance in the active avoidance task was significantly impaired following a CHI (*p*_(injury)_: avoidance = 0.003, latency = 0.0023; repeated-measures ANOVA). This impairment was consistent across age groups, but there was a significant effect of age (*p*_(age)_: avoidance = 0.017, latency = 0.014; repeated-measures ANOVA). In the active avoidance task, no age-by-injury interaction was observed in either females or males. The observation that females showed more pronounced CHI-induced deficits must be considered alongside the overall poorer task performance seen in males, which likely influenced the smaller effects observed in the male mice.

### Astrocytes are hyporesponsive and show signs of degeneration in aged animals after a CHI

**FIG. 5.**
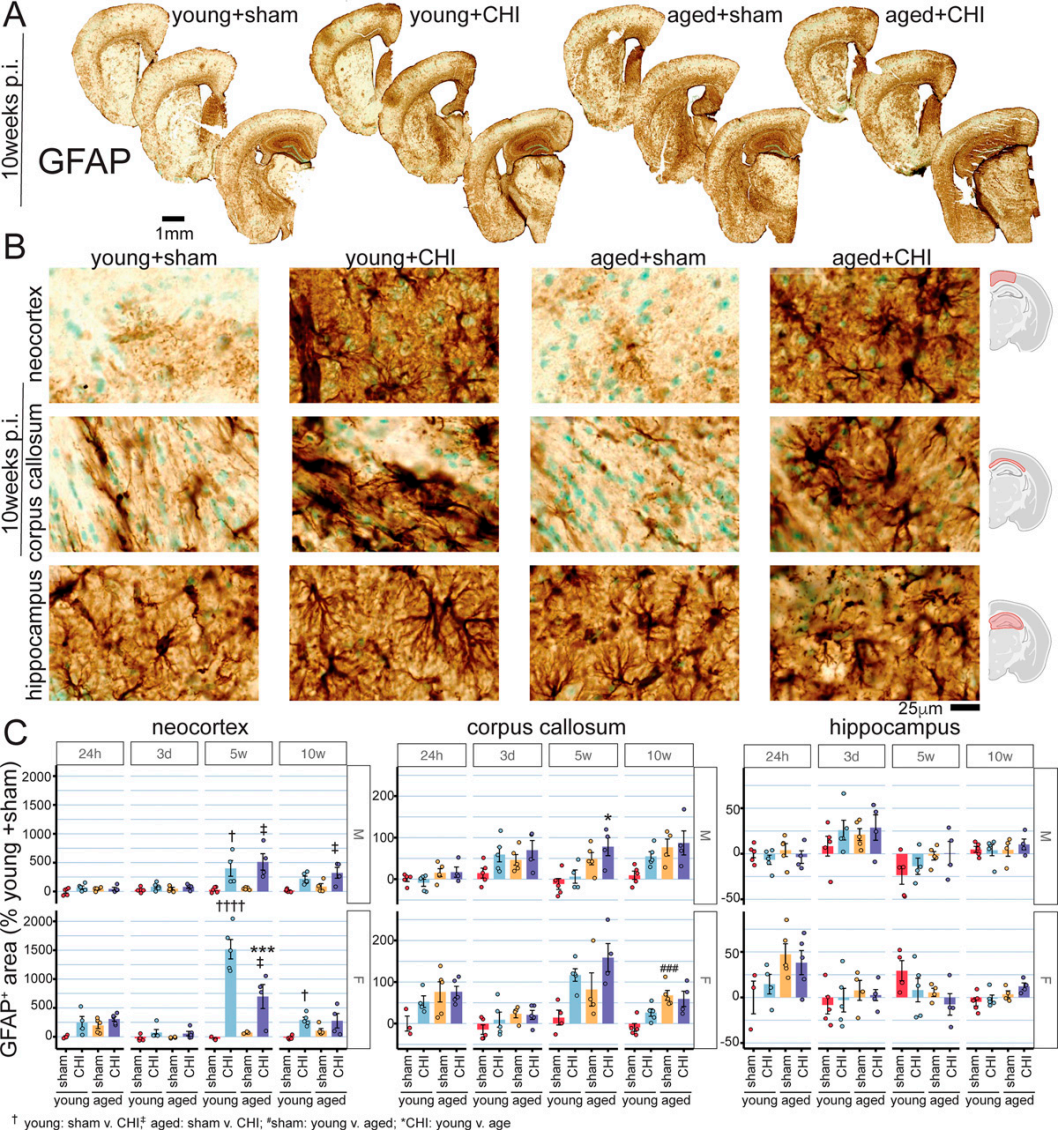
Temporal assessment of GFAP staining postinjury. Representative photomicrographs illustrate GFAP staining in various brain regions **(A)**, with detailed images **(B)** highlighting astrocytic morphological changes at 10 weeks postinjury. In the hippocampus of aged+CHI mice, images reveal beading and fragmented processes, indicative of potential dystrophic changes occurring chronically postinjury in aged mice. Digital pathological quantification of GFAP staining area averaged across 6–9 brain sections per subject was conducted for the neocortex **(C)**, corpus callosum **(D)**, and hippocampus **(E)**, encompassing the entire region as outlined in (B). The quantification shows no signs of aging intensifying reactive astrocyte changes. The results suggest, particularly in female mice, that astrocytes in aged animals are less responsive to injury than younger counterparts. Data are presented as mean ± SEM, with circles representing individual animals. Symbols denote significance as determined by a Tukey test. Significance levels: *p < 0.05; **p < 0.01; ***p < 0.005; ****p < 0.001 (*n* = 4–5 per group). CHI, closed-head injury; GFAP, glial fibrillary acidic protein.

Reactive astrocytes were assessed in the neocortex, corpus callosum, and hippocampus using GFAP immunohistochemical staining. The evaluation covered both acute (at 24 h and 3 days) and chronic (at 5 and 10 weeks) postinjury time points (Fig. 5).

In the neocortex, CHI was linked to increased GFAP-positive staining, particularly at 5 weeks postinjury (5w, male *p*_(injury)_ = 0.0003; 5w, female *p*_(injury)_ < 0.0001, two-way ANOVA) (Fig. 5C). A significant age-by-injury interaction occurred in females (*p*_(interaction)_ = 0.0058, two-way ANOVA), where aged+CHI females exhibited less GFAP staining compared with young+CHI females (*p* = 0.0048, Tukey test). By 10 weeks postinjury, there are indications of diminishing reactive astrocyte changes from the 5-week peak. However, in females, astrocyte reactivity remained heightened after CHI (10w, female *p*_(injury)_ = 0.0013, two-way ANOVA), with the young+CHI group demonstrating greater GFAP staining than the young+sham group (*p* = 0.022, Tukey test).

In the corpus callosum (Fig. 5D), GFAP staining showed an age-related increase in both females (24 h, *p*_(age)_ = 0.022; 10w, *p*_(age)_ = 0.0004, two-way ANOVA) and males (24 h, *p*_(age)_ = 0.038; 5w, *p*_(age)_ = 0.0011; 10w, *p*_(age)_ = 0.014, two-way ANOVA). In young+CHI females, increased GFAP staining was observed at 5 weeks postinjury, a trend not seen in young+CHI males (females, 5w, *p*_(injury)_ = 0.0062, two-way ANOVA).

In the hippocampus, characterized by high GFAP expression, the total staining amount remained unchanged (Fig. 5E). Yet, in the aged+CHI groups, 10 weeks postinjury, astrocytes presented with a dystrophic morphology associated with beaded and fragmented processes, similar to those seen in chronic traumatic encephalopathy (CTE),^31^ suggesting the possibility of degenerative astrocytic changes in aged mice (Fig. 5B). Future studies are needed to quantify the extent of the dystrophic astrocytic changes with age and injury.

### Injury-induced IBA1 staining displays age-dependent resolution deficits, most pronounced in the corpus callosum, alongside sex-specific differences in the neocortex and hippocampus

**FIG. 6.**
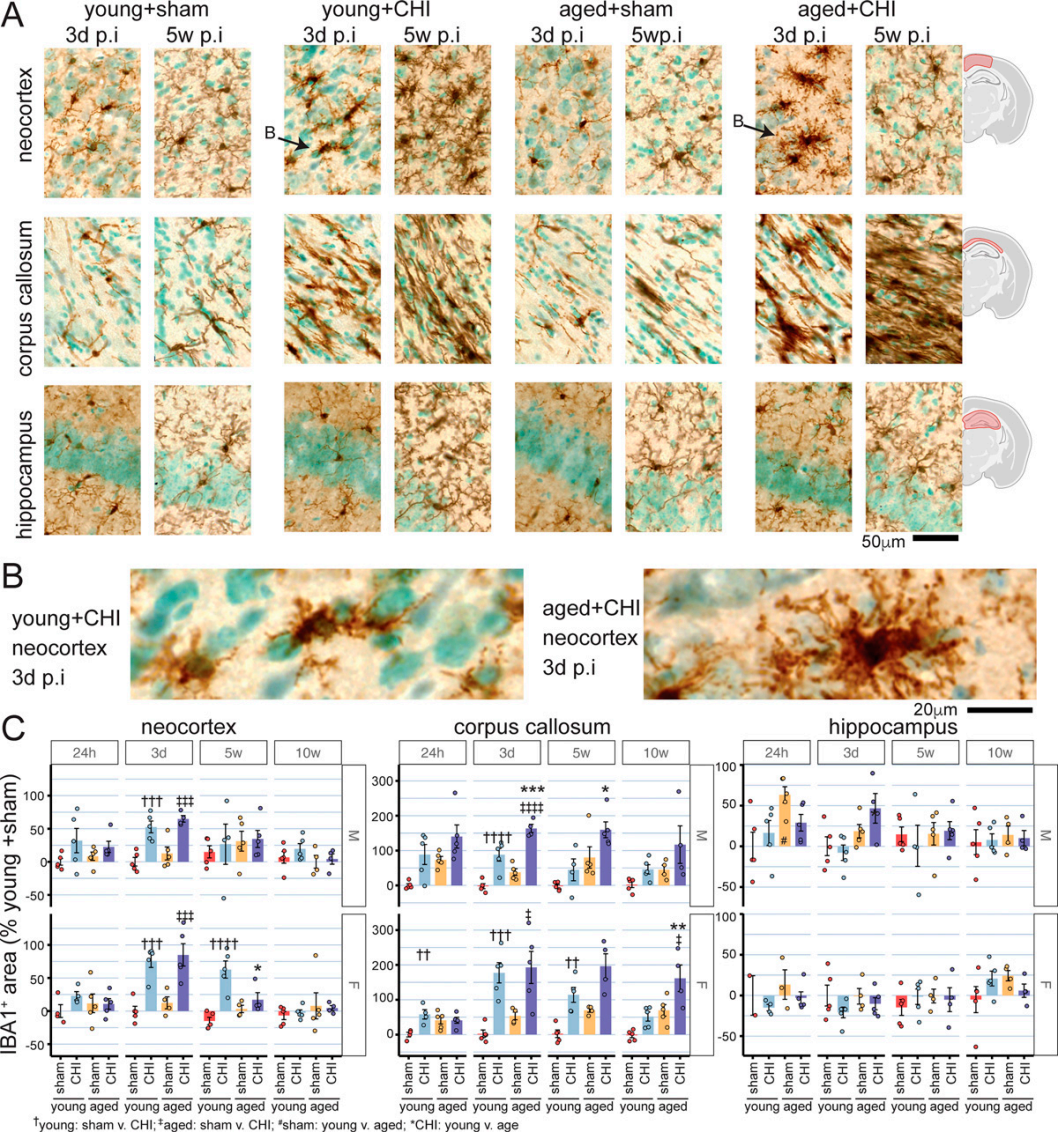
Postinjury temporal profile of IBA1 staining. **(A)** Representative photomicrographs illustrate IBA1 staining in the neocortex, corpus callosum, and hippocampus at 3 days and 5 weeks postinjury in female mice. The arrow in (A) points to cells shown at higher magnification in **(B)**, highlighting the difference in IBA1 morphology between young and aged mice at 3 days postinjury, with the IBA1^+^ cell in the aged animal showing elaborate arborization with many phagocytic cups. Digital pathological quantification of IBA1 staining area averaged across 6–9 brain sections per subject was conducted for the neocortex **(C)**, corpus callosum **(D)**, and hippocampus **(E)**, encompassing the entire region as outlined in (A). The data were normalized to the overall average of all male and female young sham mice at each time point and are presented as a percentage change from the sham. Both age- and sex-dependent effects on the microglial response to brain injury were observed. Aged mice exhibit a diminished capacity to resolve injury-induced inflammation compared with young mice, particularly in the corpus callosum. Sex-specific differences were observed in the neocortex and hippocampus. Data are presented as mean ± SEM, with circles representing individual animals. Symbols denote significance as determined by a Tukey test. Significance levels: *p < 0.05; **p < 0.01; ***p < 0.005; ****p < 0.001 (*n* = 4–5 per group). CHI, closed-head injury.

We evaluated the effects of age and injury on microglia and macrophages using IBA1 (Fig. 6) staining in the neocortex, corpus callosum, and hippocampus. IBA1 staining revealed a peak response to injury at 3 days postinjury in both male and female mice (male *p*_(injury)_<0.0001; female *p*_(injury)_<0.0001, two-way ANOVA) (Fig. 6A–C). While overall IBA1^+^ area increases were similar in young and aged mice with injury, the cells appeared morphologically different. IBA1^+^ cells from aged+CHI mice show highly complex arborization with many apparent phagocytic cups, not seen in young+CHI mice (Fig. 6B), suggesting a potentially enhanced phagocytic activity.

In males, injury-induced IBA1 changes resolved by 5 weeks. However, in the young+CHI female, the injury-induced increase in IBA1 was persistent at 5 weeks postinjury (female *p*_(injury)_ = 0.0003, two-way ANOVA) (Fig. 6C), highlighting a sex-specific difference in the inflammatory response. In addition, there was an interaction between age and injury in females, with aged+CHI mice showing less injury-induced microglial reactivity at 5 weeks postinjury than young+CHI mice (*p*_(interaction)_ = 0.0049, two-way ANOVA). By 10 weeks postinjury, the effects of age, injury, and their interaction were no longer observed, indicating a resolution of injury-induced changes of IBA1^+^ cells within the neocortex across all groups.

In the corpus callosum (Fig. 6A, D), the CHI led to greater IBA1 staining across all time points in both female and male mice (*p*_(injury)_; female 24 h = 0.014, 3d < 0.001, 5w < 0.001, 10w = 0.002; male 24 h = 0.003, 3d < 0.001, 5w = 0.02, 10w = 0.043; two-way ANOVA). Aging was also associated with more IBA1 staining (*p*_(age)_; female 5w = 0.003, 10w = 0.0003; male 24 h = 0.014, 3d = 0.0003, 5w = 0.001, 10w = 0.046; two-way ANOVA). Young mice exhibited a peak in injury-induced IBA1 changes at 3 days postinjury. These changes resolved over 5 weeks, with IBA1 levels returning to sham levels in both sexes by 10 weeks postinjury. Conversely, aged+CHI mice (both male and female) displayed consistent IBA1 staining from 3 days to 10 weeks postinjury. This suggests ongoing inflammation and impaired resolution of the injury-induced changes. A significant age-by-injury interaction was observed in female mice at 24 h postinjury, with young+CHI mice demonstrating an injury-induced increase in IBA1 staining, while aged+CHI mice did not (*p*_(interaction)_ = 0.013, two-way ANOVA).

Female mice showed no hippocampal microglial changes related to injury, age, or their interaction (Fig. 6A, E). In contrast, male mice exhibited age-dependent increases in hippocampal IBA1 staining at 24 h and 3 days postinjury (*p*_(age)_; male 24 h = 0.013, 3d = 0.007; two-way ANOVA). However, no effects of injury or age-by-injury interactions were observed in males.

### Injury-induced CD45 histopathology reveals age-dependent increases in the corpus callosum and age-dependent hyporesponsiveness in the neocortex following CHI

**FIG. 7.**
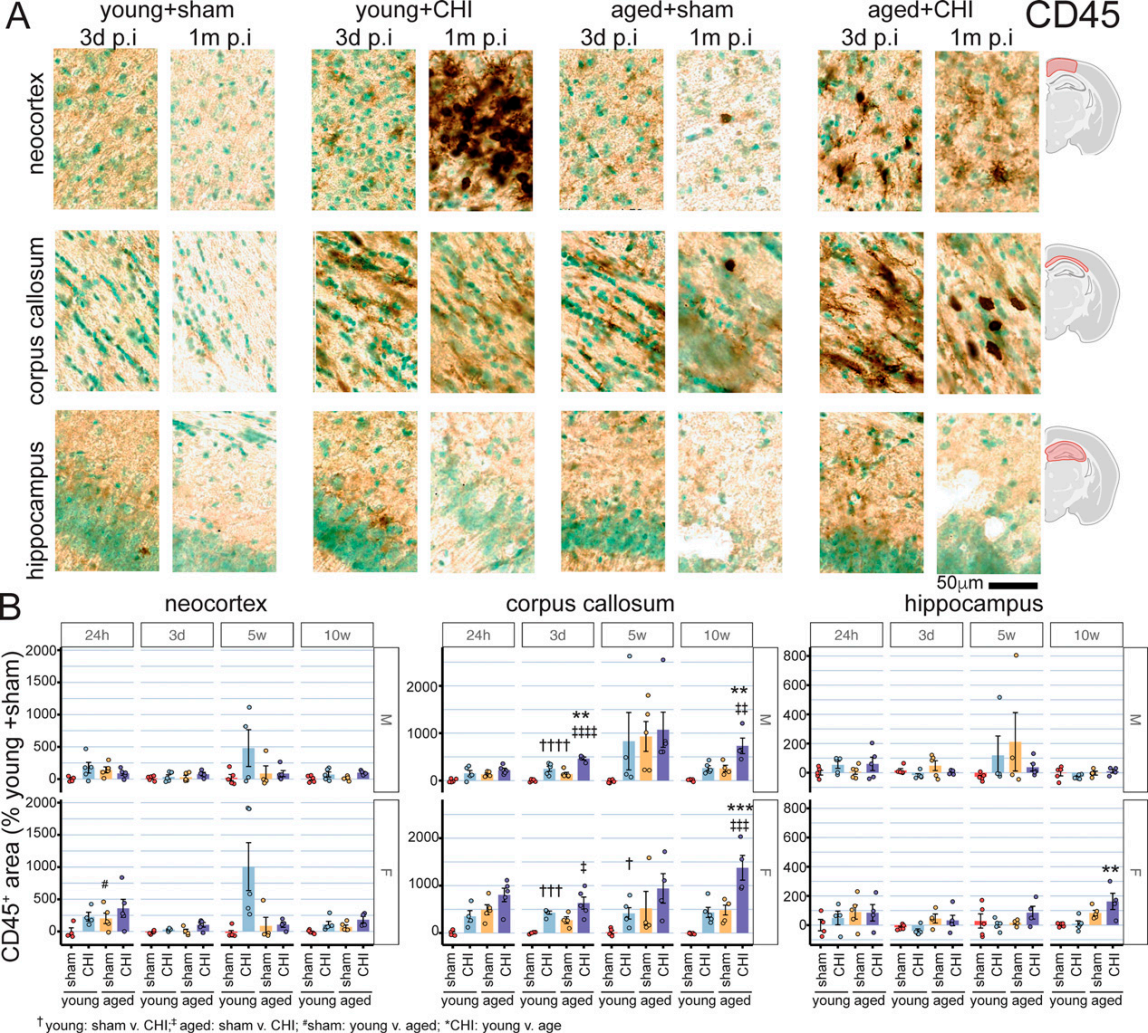
Temporal changes in CD45 staining postinjury**. (A)** CD45 staining in the neocortex, corpus callosum, and hippocampus at 3 days and 5 weeks postinjury. The arrows indicate lymphocyte-like morphology in the aged mouse corpus callosum. **(B)** Young+CHI mice show increased neocortical CD45 staining (female *p*_(interaction)_ = 0.024). **(C)** Aged+CHI female mice exhibit the greatest increase in corpus callosum CD45, with age-by-injury interactions at most time points, except at 24 h (*p*_(interaction)_ < 0.05). Male mice show similar trends with a reduced magnitude. **(D)** Few CD45+ cells were seen in the hippocampus. Data: mean ± SEM, with circles representing individual animals. Symbols: Tukey test, significance levels: *p < 0.05; **p < 0.01; ***p < 0.005; ****p < 0.001. The sample size is 4–5 per group. CHI, closed-head injury.

CD45 (lymphocyte common antigen), a marker expressed at low levels in the uninjured brain but increased with injury, was used to evaluate inflammatory responses. At 5 weeks postinjury, robust CD45 staining was localized in the neocortex, primarily within the brain area closest to the CHI impact zone (Fig. 7A). This effect was most apparent in female young+CHI mice (Fig. 7B) (*p*_(interaction)_ = 0.024, two-way ANOVA). In the neocortex of female mice, injury-induced increases in CD45 staining were found at all time points (*p*_(injury)_; 24 h = 0.02, 3d = 0.008, 5w = 0.0009, 10w = 0.0015; two-way ANOVA). In contrast, in the neocortex, injury-induced CD45 staining was observed in male mice only at 10 weeks postinjury (*p*_(injury)_ = 0.026; two-way ANOVA). In males, an injury-by-age interaction occurred at 24 h postinjury in the cortex; young+CHI mice showed greater CD45 staining than their age-matched sham counterparts, but this effect was not seen in aged+CHI mice (male 24 h; *p*_(interaction)_ = 0.034, two-way ANOVA). These findings suggest that sex and age significantly influence the timing and intensity of the neocortical inflammatory response following brain injury, with females demonstrating a more rapid and sustained reaction than males, and evidence of age-related weakening of the early inflammatory response.

Changes in CD45 by age and injury were prominent in the corpus callosum (Fig. 7C). Both female and male mice demonstrated increased CD45 staining in response to injury (*p*_(injury)_; female 24 h = 0.0009, 3d < 0.0001, 5w = 0.0048, 10w < 0.0001; male 24 h = 0.0084, 3d < 0.0001, 5w = 0.012, 10w < 0.0001; two-way ANOVA). This injury-induced increase in CD45 staining was similar in magnitude and temporal pattern for both sexes, with levels rising from 24 h to 10 weeks postinjury. This suggests that chronic degeneration in the corpus callosum is driving progressive inflammatory cell engagement.

In the corpus callosum, aged mice, both male and female, exhibited greater CD45 staining at all postinjury time points (*p*_(age)_; female 24 *h* < 0.0001, 3d < 0.0001, 5w = 0.0077, 10w < 0.0001; male 24 h = 0.0049, 3d < 0.0001, 5w = 0.0009, 10w < 0.0001; two-way ANOVA). Aged+CHI female mice showed the most significant increase in CD45 staining, with age-by-injury interactions observed at all time points except 24 h postinjury (*p*_(interaction)_; 3d = 0.004, 5w = 0.031, 10w = 0.042; two-way ANOVA). In males, age-by-injury interactions were seen only at 5 weeks postinjury (*p*_(interaction)_ = 0.042).

In addition, at 10 weeks postinjury, the morphological appearance of cells stained for CD45 differed with age in aged+CHI mice. Many cells had a round, lymphocyte-like appearance, contrasting with the ramified, microglia-like appearance in young+CHI mice (Fig. 7A). Even without injury, aged+sham mice displayed round, lymphocyte-like cells in the corpus callosum (Fig. 7A) suggesting an age-associated CD45 phenotype.

Few CD45^+^ cells were seen in the hippocampus. No effect of injury or age-by-injury interaction was seen in the male or female mice at any time point postinjury. An effect of age was seen in the female mice at 3 days and 10 weeks postinjury (*p*_(age)_; 3d = 0.011, 10w = 0.0002).

### A sexually dimorphic interaction of age and injury is observed with the disease-associated microglia marker dectin-1, with young female mice exhibiting the most significant staining following a CHI

**FIG. 8.**
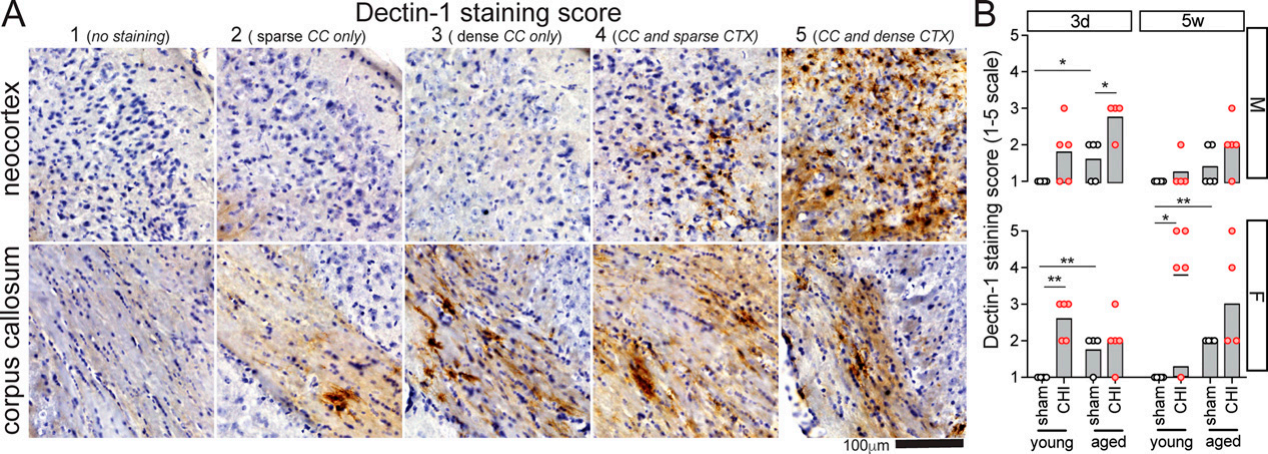
Temporal changes in dectin-1 staining post-injury. **(A)** This figure presents an example of the pattern of Dectin-1 staining observed in the neocortex (CTX) and corpus callosum (CC), which was used to establish the pathological staining score. The scale is defined as follows: 1 = none, 2 = sparse in corpus callosum, 3 = dense in corpus callosum, 4 = sparse in both cortex and corpus callosum, and 5 = dense in both cortex and corpus callosum. The neocortex and corpus callosum images of each example score are from the same animal. Dectin-1 staining in the neocortex was not observed without concurrent staining in the corpus callosum. No Dectin-1 staining was observed in the hippocampus**. (B)** The highest Dectin-1 staining score was observed in young+CHI female mice. While an effect of injury was noted in both males and females at 3 days postinjury (*p*_(injury)_ < 0.001), by 5 weeks, injury-related changes had resolved in male mice but had intensified in female mice (*p*_(injury)_ = 0.0001). Data are presented as median, with circles representing individual animals. Symbols: Chi-square test, significance levels: *p < 0.05; **p < 0.01; ***p < 0.005; ****p < 0.001. The sample size is 4–5 per group. CHI, closed-head injury.

Dectin-1, also known as Clec7a, is a pattern-recognition receptor that recognizes polysaccharides (Beta-1,3 and Beta-1,6 glucans). Serving as a marker of disease-associated microglia, it has previously been linked to exacerbated inflammation and gliosis after TBI in aged animals.^23,32^ Three days postinjury, Dectin-1 expression was observed in a few sparse cells within the corpus callosum (Fig. 8A). By five weeks postinjury, Dectin-1 staining was predominantly found in the corpus callosum, with some animals showing staining in the cortex. While Dectin-1 staining was generally sparse, with only a few positive cells seen across most animals, two young+CHI female animals exhibited dense staining near the primary impact area of the cortex. In males, greater Dectin-1 staining correlated with both age and injury (*p*_(age)_ 3d = 0.002, 5w = 0.01; *p*_(injury)_ 3d = 0.0006; two-way logistic regression), with aged+CHI mice displaying the highest Dectin-1 staining scores among male groups (Fig. 8A). For females, age, injury, and interaction effects were significant (*p*_(age)_ 5w = 0.049; *p*_(injury)_ 3d = 0.0005, 5w = 0.0001; *p*_(interaction)_ 3d = 0.004, 5w = 0.007; two-way logistic regression) (Fig. 8A). Unlike males, young+CHI female mice demonstrated the highest level of Dectin-1 staining among all groups.

## Discussion

Our results in mice following a mild CHI find that aged females exhibit the lowest phenotypic recovery from the injury, as evidenced by their poor performance on the RAWM and active avoidance tasks of memory. A worse behavioral response to injury has been reported in male animals following an open-skull moderate-to-severe TBI.^11,13,18,19,22,23^ However, as previous studies have only used male mice or did not report the sex of the animals used, it is currently unknown if female mice would also exhibit worse outcomes following an open-skull moderate-to-severe TBI.

Pathologically, we find the greatest interaction of age by injury in the corpus callosum, with aged mice, particularly aged female mice, showing greater pathological markers of neuroinflammation. This suggests that white matter tracts may be particularly vulnerable to the effects of injury with age. Indeed, in aged mice, after injury, there is reduced oligodendrogenesis.^14^ The failure to replace lost oligodendrocytes and to repair myelin could contribute to impaired behavioral outcomes and chronic inflammation in the white matter. A single CHI in young adult animals causes myelin damage and disorganization of the nodes of Ranvier.^33^ It is yet unknown how aging affects myelin integrity and organization of the nodes of Ranvier; however, our work highlights the need for more research focused on the mechanisms of age-related white matter vulnerability.

In contrast to prior open-skull moderate-to-severe TBI studies, which found greater astrocyte reactivity after injury in aged animals,^10,20,23,34^ our pathological quantification showed comparable GFAP expression magnitudes between young and aged animals. In male animals, we found little evidence of an age-by-injury interaction. However, we observed this interaction in female animals at 5 weeks postinjury, with less GFAP staining in aged+CHI mice than young+CHI mice. This is unexpected, as GFAP often strongly correlates with injury severity. Yet, aged female mice exhibited the greatest phenotypic change, suggesting a more severe injury and a disconnect between phenotype and pathology. Prior work in mild closed-head TBI models has shown that failure to form astrocytic boundaries, associated with decreased GFAP expression, contributed to persistent barrier dysfunction and loss of homeostatic astrocytic responses in atypical astrocytes.^35,36^ Thus, failing to increase GFAP expression in aged animals could be a maladaptive response. In addition, we found evidence of astrocyte degeneration in the hippocampus of aged mice after CHI, mirroring CTE.^31^ Notably, our previous findings demonstrate distinctive aging-associated astrocytic changes in the hippocampus, including enlarged soma and vacuolization of processes characteristic of clasmatodendrosis.^10,37^

Microglia-mediated neuroinflammation has been strongly linked to worse outcomes after open-skull moderate-to-severe TBI in aged animals,^18,23^ with several studies demonstrating a greater reactive microglia response in aged versus young-adult animals.^7,13,17–20,23^ Our results in the corpus callosum align with these prior studies, finding greater IBA1 and CD45 staining in injured aged animals compared with young-adult animals. However, our findings that microglia in aged female mice are less responsive to injury than their younger counterparts highlight differences that may be associated with a unique age-by-sex interaction not previously evaluated or regional tissue vulnerability in aged animals not previously identified. While most studies showed greater neuroinflammation after this injury, one study found less neuroinflammation in the contralesionally hemisphere of aged animals compared with young adult injured animals after a controlled cortical impact.^22^ These results further support that the aged brain may be hyporesponsive to some insults, which could depend on age-associated alterations to the tissue environment and cell-type-specific changes in microglia. These changes include the accumulation of senescent and dystrophic microglia, which have been shown to be significant contributors to the inflammation seen after TBI in aged animals,^18^ and also linked to a disease-associated phenotype in people.^38^ Although the amount of microglia staining was comparable between the young and aged animals after injury, there were some morphological differences, including the highly ramified IBA1 cells seen in the injured-aged animals. We speculated that these ramified cells could be associated with a disease-associated microglia phenotype, identified by clec7a/Dectin1. However, we found little evidence to support this conclusion, as Dectin1 staining was absent from the cortex of most aged animals at 5 weeks postinjury, suggesting that these highly ramified cells were not Dectin1-positive cells.

Previous studies have shown increased type I and II interferon signaling in aged animals after a TBI,^7,23^ which can modulate lymphocyte activation, differentiation, and migration (type I) and are predominantly produced by T cells (type II). Ritzel et al. found an expansion of the CD45hiCD11b-lymphocyte pool in aged animals following an open-skull moderate-to-severe TBI.^17^ Aged animals exhibit an increased T cell signature after an open-skull moderate-to-severe TBI in the meninges,^8^ and type II interferon-producing T cells are known to increase in the meninges of aged animals.^39^ In agreement with these prior studies, we found round lymphocyte-like CD45+ cells particularly enriched in aged animals. While we do not yet know the cellular identity of these CD45+ cells, they appear to be a unique age-associated phenotype not present in young animals.

The limitation of our study is that it may be underpowered to detect certain age-by-injury interactions reliably. Our power analysis was based on expecting a stronger interaction effect and a more pronounced phenotype than we observed. Despite this, we see clear evidence of age-by-injury interactions, even if these did not always reach statistical significance given our sample size. These results provide a strong foundation for future studies, which should delve deeper into understanding the mechanisms that increase the aged brain’s vulnerability to negative TBI impacts and subsequently develop therapies tailored to accelerate recovery and rehabilitation after injury.

## Data Availability

All data are publicly available in the open data commons for traumatic brain injury (ODC-TBI) at the following DOI link: https://doi:10.34945/F5TP4F

## Abbreviations Used

AUC: area under the curve
ANOVA: analysis of variance
CHI: closed-head injury
Clec7a: C-type lectin domain family 7 member A
GFAP: glial fibrillary acidic protein
IBA1: ionized calcium binding adapter molecule 1
IHC: immunohistochemistry
mTBI: mild traumatic brain injury
SEM: standard error of the mean
TBI: traumatic brain injury

## References

[1] Albrecht JS, Slejko JF, Stein DM, et al. Treatment Charges for Traumatic Brain Injury Among Older Adults at a Trauma Center. J Head Trauma Rehabil 2017;32(6):E45–E53; doi: 10.1097/HTR.0000000000000297 28195959 PMC5552449

[2] Bailey MD, Gambert S, Gruber-Baldini A, et al. Traumatic Brain Injury and Risk of Long-Term Nursing Home Entry among Older Adults: An Analysis of Medicare Administrative Claims Data. J Neurotrauma 2023;40(1–2):86–93; doi: 10.1089/neu.2022.0003 35793112 PMC10162579

[3] Dams-O’Connor K, Cuthbert JP, Whyte J, et al. Traumatic brain injury among older adults at level I and II trauma centers. J Neurotrauma 2013;30(24):2001–2013; doi: 10.1089/neu.2013.3047 23962046 PMC3868380

[4] Forssten SP, Ahl Hulme R, Forssten MP, et al. Predictors of outcomes in geriatric patients with moderate traumatic brain injury after ground level falls. Front Med (Lausanne) 2023;10:1290201; doi: 10.3389/fmed.2023.1290201 38152301 PMC10751787

[5] Gardner RC, Dams-O’Connor K, Morrissey MR, et al. Geriatric Traumatic Brain Injury: Epidemiology, Outcomes, Knowledge Gaps, and Future Directions. J Neurotrauma 2018;35(7):889–906; doi: 10.1089/neu.2017.5371 29212411 PMC5865621

[6] Winter L, Mensinger JL, Moriarty HJ, et al. Age Moderates the Effect of Injury Severity on Functional Trajectories in Traumatic Brain Injury: A Study Using the NIDILRR Traumatic Brain Injury Model Systems National Dataset. J Clin Med 2022;11(9); doi: 10.3390/jcm11092477 PMC9104127 35566607

[7] Barrett JP, Knoblach SM, Bhattacharya S, et al. Traumatic Brain Injury Induces cGAS Activation and Type I Interferon Signaling in Aged Mice. Front Immunol 2021;12:710608; doi: 10.3389/fimmu.2021.710608 34504493 PMC8423402

[8] Buenaventura RG, Harvey AC, Burns MP, et al. Traumatic brain injury induces an adaptive immune response in the meningeal transcriptome that is amplified by aging. Front Neurosci 2023;17:1210175; doi: 10.3389/fnins.2023.1210175 37588516 PMC10425597

[9] Criado-Marrero M, Ravi S, Bhaskar E, et al. Age dictates brain functional connectivity and axonal integrity following repetitive mild traumatic brain injuries. bioRxiv 2024; doi: 10.1101/2024.01.25.577316 PMC12083070 39089604

[10] Early AN, Gorman AA, Van Eldik LJ, et al. Effects of advanced age upon astrocyte-specific responses to acute traumatic brain injury in mice. J Neuroinflammation 2020;17(1):115; doi: 10.1186/s12974-020-01800-w 32290848 PMC7158022

[11] Islam MBAR, Davis BT, Kando MJ, et al. Differential neuropathology and functional outcome after equivalent traumatic brain injury in aged versus young adult mice. Exp Neurol 2021;341:113714; doi: 10.1016/j.expneurol.2021.113714 33831399 PMC8276249

[12] Krukowski K, Chou A, Feng X, et al. Traumatic Brain Injury in Aged Mice Induces Chronic Microglia Activation, Synapse Loss, and Complement-Dependent Memory Deficits. Int J Mol Sci 2018;19(12); doi: 10.3390/ijms19123753 PMC6321529 30486287

[13] Kumar A, Stoica BA, Sabirzhanov B, et al. Traumatic brain injury in aged animals increases lesion size and chronically alters microglial/macrophage classical and alternative activation states. Neurobiol Aging 2013;34(5):1397–1411; doi: 10.1016/j.neurobiolaging.2012.11.013 23273602 PMC3572914

[14] Michalettos G, Clausen F, Özen I, et al. Impaired oligodendrogenesis in the white matter of aged mice following diffuse traumatic brain injury. GLIA 2024;72(4):728–747; doi: 10.1002/glia.24499 38180164

[15] Onyszchuk G, He YY, Berman NE, et al. Detrimental effects of aging on outcome from traumatic brain injury: A behavioral, magnetic resonance imaging, and histological study in mice. J Neurotrauma 2008;25(2):153–171; doi: 10.1089/neu.2007.0430 18260798

[16] Portbury SD, Hare DJ, Sgambelloni CJ, et al. Age modulates the injury-induced metallomic profile in the brain. Metallomics 2017;9(4):402–410; doi: 10.1039/c6mt00260a 28170006

[17] Ritzel RM, Doran SJ, Glaser EP, et al. Old age increases microglial senescence, exacerbates secondary neuroinflammation, and worsens neurological outcomes after acute traumatic brain injury in mice. Neurobiol Aging 2019;77:194–206; doi: 10.1016/j.neurobiolaging.2019.02.010 30904769 PMC6486858

[18] Ritzel RM, Li Y, Jiao Y, et al. Brain injury accelerates the onset of a reversible age-related microglial phenotype associated with inflammatory neurodegeneration. Sci Adv 2023;9(10):eadd1101; doi: 10.1126/sciadv.add1101 36888713 PMC9995070

[19] Ritzel RM, Li Y, Lei Z, et al. Functional and transcriptional profiling of microglial activation during the chronic phase of TBI identifies an age-related driver of poor outcome in old mice. Geroscience 2022;44(3):1407–1440; doi: 10.1007/s11357-022-00562-y 35451674 PMC9213636

[20] Sandhir R, Onyszchuk G, Berman NE. Exacerbated glial response in the aged mouse hippocampus following controlled cortical impact injury. Exp Neurol 2008;213(2):372–380; doi: 10.1016/j.expneurol.2008.06.013 18692046 PMC2662478

[21] Timaru-Kast R, Herbig EL, Luh C, et al. Influence of Age on Cerebral Housekeeping Gene Expression for Normalization of Quantitative Polymerase Chain Reaction after Acute Brain Injury in Mice. J Neurotrauma 2015;32(22):1777–1788; doi: 10.1089/neu.2014.3784 26102571

[22] Timaru-Kast R, Luh C, Gotthardt P, et al. Influence of age on brain edema formation, secondary brain damage and inflammatory response after brain trauma in mice. PLoS One 2012;7(8):e43829; doi: 10.1371/journal.pone.0043829 22952778 PMC3431406

[23] Wangler LM, Bray CE, Packer JM, et al. Amplified Gliosis and Interferon-Associated Inflammation in the Aging Brain following Diffuse Traumatic Brain Injury. J Neurosci 2022;42(48):9082–9096; doi: 10.1523/jneurosci.1377-22.2022 36257689 PMC9732830

[24] Dutta S, Sengupta P. Men and mice: Relating their ages. Life Sci 2016;152:244–248; doi: 10.1016/j.lfs.2015.10.025 26596563

[25] Macheda T, Roberts K, Bachstetter AD. Electromagnetic Controlled Closed-Head Model of Mild Traumatic Brain Injury in Mice. J Vis Exp 2022;187; doi: 10.3791/64556 PMC10550048 36279529

[26] Macheda T, Roberts KN, Morganti JM, et al. Optimization and validation of a modified radial-arm water maze protocol using a murine model of mild closed head traumatic brain injury. PLoS One 2020;15(8):e0232862; doi: 10.1371/journal.pone.0232862 32810143 PMC7433858

[27] Macheda T, Snider HC, Watson JB, et al. An active avoidance behavioral paradigm for use in a mild closed head model of traumatic brain injury in mice. J Neurosci Methods 2020;343:108831; doi: 10.1016/j.jneumeth.2020.108831 32592717 PMC7418983

[28] Bachstetter AD, Norris CM, Sompol P, et al. Early stage drug treatment that normalizes proinflammatory cytokine production attenuates synaptic dysfunction in a mouse model that exhibits age-dependent progression of Alzheimer’s disease-related pathology. J Neurosci 2012;32(30):10201–10210; doi: 10.1523/JNEUROSCI.1496-12.2012 22836255 PMC3419360

[29] Bachstetter AD, Rowe RK, Kaneko M, et al. The p38alpha MAPK regulates microglial responsiveness to diffuse traumatic brain injury. J Neurosci 2013;33(14):6143–6153; doi: 10.1523/JNEUROSCI.5399-12.2013 23554495 PMC3667712

[30] Rowe RK, Ellis GI, Harrison JL, et al. Diffuse traumatic brain injury induces prolonged immune dysregulation and potentiates hyperalgesia following a peripheral immune challenge. Mol Pain 2016;12; doi: 10.1177/1744806916647055 PMC4955995 27178244

[31] Hsu ET, Gangolli M, Su S, et al. Astrocytic degeneration in chronic traumatic encephalopathy. Acta Neuropathol 2018;136(6):955–972; doi: 10.1007/s00401-018-1902-3 30194648

[32] Deczkowska A, Keren-Shaul H, Weiner A, et al. Disease-Associated Microglia: A Universal Immune Sensor of Neurodegeneration. Cell 2018;173(5):1073–1081; doi: 10.1016/j.cell.2018.05.003 29775591

[33] Radomski KL, Zi X, Lischka FW, et al. Acute axon damage and demyelination are mitigated by 4-aminopyridine (4-AP) therapy after experimental traumatic brain injury. Acta Neuropathol Commun 2022;10(1):67; doi: 10.1186/s40478-022-01366-z 35501931 PMC9059462

[34] Moro F, Pischiutta F, Portet A, et al. Ageing is associated with maladaptive immune response and worse outcome after traumatic brain injury. Brain Commun 2022;4(2):fcac036; doi: 10.1093/braincomms/fcac036 35350551 PMC8947244

[35] George KK, Heithoff BP, Shandra O, et al. Mild Traumatic Brain Injury/Concussion Initiates an Atypical Astrocyte Response Caused by Blood-Brain Barrier Dysfunction. J Neurotrauma 2022;39(1–2):211–226; doi: 10.1089/neu.2021.0204 34806422 PMC8785769

[36] Shandra O, Winemiller AR, Heithoff BP, et al. Repetitive Diffuse Mild Traumatic Brain Injury Causes an Atypical Astrocyte Response and Spontaneous Recurrent Seizures. J Neurosci 2019;39(10):1944–1963; doi: 10.1523/JNEUROSCI.1067-18.2018 30665946 PMC6407295

[37] Lee E, Jung YJ, Park YR, et al. A distinct astrocyte subtype in the aging mouse brain characterized by impaired protein homeostasis. Nat Aging 2022;2(8):726–741; doi: 10.1038/s43587-022-00257-1 37118130

[38] Shahidehpour RK, Higdon RE, Crawford NG, et al. Dystrophic microglia are associated with neurodegenerative disease and not healthy aging in the human brain. Neurobiol Aging 2021;99:19–27; doi: 10.1016/j.neurobiolaging.2020.12.003 33422891 PMC8293930

[39] Rustenhoven J, Pavlou G, Storck SE, et al. Age-related alterations in meningeal immunity drive impaired CNS lymphatic drainage. J Exp Med 2023;220(7); doi: 10.1084/jem.20221929 PMC10083715 37027179

